# Mini-percutaneous nephrolithotomy in a child with multiple urogenital anomalies and a solitary duplex kidney

**DOI:** 10.1590/S1677-5538.IBJU.2019.0545

**Published:** 2021-02-03

**Authors:** Mehmet Çaglar Çakici, Ferhat Keser, Ramazan Gokhan Atis, Asif Yildirim

**Affiliations:** 1 Istanbul Medeniyet University Goztepe Training and Research Hospital Department of Urology Istanbul Turkey Department of Urology, Istanbul Medeniyet University Goztepe Training and Research Hospital, Istanbul, Turkey

## Abstract

**Purpose:**

To report a case of successful removal of right staghorn renal calculi in a 3-year-old girl with Arnold-Chiari malformation and multiple urogenital anomalies.

**Case report:**

A 3-year-old female child with the diagnosis of Arnold-Chiari type 2 malformation was referred to our clinic due to presence of 9 kidney stones with a total volume of 10743mm3. The total of the longest diameters of all stones was calculated as 11.4cm. The patient had a urogenital septum, bifid bladder, and duplicated collecting system on the right side. An 18F Amplatz sheath was placed and mini-percutaneous nephrolithotomy was performed successfully by laser and pneumatic lithotripter. Any residual urinary tract stones or urinary tract infection were not detected during the 6th-month follow-up.

**Conclusion:**

Urolithiasis requires a thorough understanding of the underlying causes, as well as an effective and minimally invasive treatment. It is important for urologists to understand the complexity of the optimal stone management in pediatric patients in order to maximize treatment efficacy and minimize morbidity. We conclude that it is essential to treat urolithiasis in a single session in children with urogenital anomalies and accompanying congenital anomalies who have past surgical history.

## INTRODUCTION

Urolithiasis in the pediatric population represents a significant challenge due to difficulties in diagnosis and treatment. The incidence of urolithiasis has increased over the last two decades. There are many theories regarding the rising incidence of pediatric urolithiasis including the increased detection of stones, changing dietary patterns, and higher rates of childhood obesity (1–6). The mean age of pediatric patients that have urolithiasis with a high risk of recurrence is about 7-8 years (7). Urogenital abnormalities and metabolic disorders have been reported as causative factors for pediatric urolithiasis (6, 7).

The presence of anatomical anomalies accompanied by some abdominal pathologies such as Arnold-Chiari malformation may affect the success of surgeries. The Chiari malformation is a congenital anomaly of the craniovertebral junction and hindbrain (8). Herein, we report a case of a 3-year-old girl with Arnold-Chiari malformation whose right staghorn renal calculi were successfully removed in a single session.

### Case Presentation

A 3-year-old female child with the diagnosis of Arnold-Chiari type 2 malformation was referred to our clinic due to recurrent urinary tract infections and voiding dysfunction.

The patient's past surgical history included surgical treatment of meningomyelocele and anal atresia during the neonatal period and cystolithotripsy procedure by laser lithotripter for two bladder stones of 15 and 20mm six months ago. The ultrasound examination of the urinary system revealed multiple stones in the right kidney with the largest one being 24mm in diameter and left-sided renal atrophy. In addition, there was grade 2 hydronephrosis of right pelvicalyceal system. An 8F percutaneous nephrostomy catheter was placed into the collecting system of the right kidney. Non-contrast low dose computed tomography showed right renal staghorn calculi with the largest one measuring 24x18mm and 1003 Hounsfield units. In total, 9 kidney stones with a total volume of 10743mm3 were detected. The sum of the longest diameters of all stones was calculated as 11.4cm. The 24-hour urine examinations showed no hypercalciuria, hyperoxaluria, hypocitraturia or cystinuria. Serum laboratory examinations showed no electrolyte imbalance or hyperparathyroidism and arterial gas analysis did not reveal acidemia or alkalemia. Renal scintigraphy with dimercaptosuccinic acid showed 98% split function for right kidney and 2% function for left kidney. Voiding cystourethrogram showed no reflux.

Mini-percutaneous nephrolithotomy was planned for right kidney stones following a sterile urine culture. A cystoscopy was performed under general anesthesia. Urogenital septum dividing the bladder and vagina was detected 1cm proximal to the urogenital opening. There was a bifid bladder with two ureteral orifices on the right side and one with a close ending ureter on the left side. A ureteroscopy was performed before positioning the patient for percutaneous nephrolithotomy. We observed that two separate collection systems drained independently to the right side of the bladder. There were no stones in the collecting system that opened to the bladder via the lower pole ureter. However, in the collecting system that opened to the bladder by the upper pole ureter, stones extending from the kidney to the ureter were observed. Afterwards, 5F ureteral catheter was placed up to the renal pelvis and the patient was placed in prone position. Before starting the procedure, the nephrostomy catheter which was inserted 3 weeks ago was removed. Percutaneous access was achieved under fluoroscopy guidance using an 18-gauge needle and guidewire and the nephrostomy tract was dilated up with polyurethane serial Amplatz dilators. An 18F Amplatz sheath was then placed over the dilators and the stones were fragmented by laser and pneumatic lithotripter. We started the fragmentation with laser lithotripsy and switched to pneumatic lithotripsy due to high stone burden and hardness. Although a residual stone of 5mm in size was seen on fluoroscopy, it could not be reached and a 3cm 8F Malecot nephrostomy tube was introduced to the right kidney. The procedure was terminated after Foley urethral catheter was inserted. The total surgical time was 105 minutes.

Nephrostomy tube was clamped on postoperative 2nd day and remained clamped on postoperative 3rd day, however urinary extravasation was detected around the nephrostomy. A diagnostic ureteroscopy was performed for the residual ureteral stone. Stone fragments visualized in the ureter were extracted by foreign body forceps and nephrostomy tube was detected at the level of ureteropelvic junction. A 12cm 4F double-J ureteral stent was placed and the procedure was completed. Double-J stent was removed cystoscopically and the nephrostomy tube was removed during the same procedure on postoperative second day under sedation. Subsequently, the patient was discharged. Within one week, the patient recovered and resumed normal life. At the 6th month follow––up, there were no residual or recurrent renal stones. Informed written consent was obtained from the patient's family regarding the publication of the case study.

## DISCUSSION

The prevalence of pediatric urolithiasis is approximately 2% and has been increasing worldwide over the last two decades (9). This increase has been related to various causes including genetic, anatomical, metabolic, dietary, and environmental factors. The success of the treatment is dependent on multiple factors, including factors that affect stone formation.

Although the exact cause of Arnold-Chiari malformation is unknown, it has been linked to abnormal brain formation during fetal development. This malformation may occur as a result of exposure to teratogenic substances during fetal development or may be related to genetic problems and syndromes with familial transmission.

Indications for percutaneous nephrolithotomy in pediatric population include large stone burden (>2cm), lower pole calculi greater than 1cm, anatomic abnormality impairing urinary drainage, and cystine or struvite stones (10). Despite initial concerns regarding the impact of this surgery on renal function, prolonged fluoroscopy time and the risk of bleeding, studies have shown no risk of renal function deterioration or renal scarring in pediatric population (11). Studies have reported stone-free rates ranging from 87% to 98.5% in children that underwent percutaneous nephrolithotomy (11–13).

## CONCLUSION

Urolithiasis is a common condition and its incidence is increasing among all demographic groups worldwide. It requires a thorough understanding of underlying causes and it is important for the urologist to understand the complexities of the optimal management in pediatric patients, in order to maximize treatment efficacy with minimal morbidity.

## References

[1] 1. Xiao B, Zhang X, Hu WG, Chen S, Li YH, Tang YZ, et al. Minipercutaneous Nephrolithotomy Under Total Ultrasonography in Patients Aged Less Than 3 Years: A Single-center Initial Experience from China. Chin Med J (Engl). 2015; 128:1596-600. 10.4103/0366-6999.158312 PMC4733741 26063360

[2] 2. Sas DJ, Hulsey TC, Shatat IF, Orak JK. Increasing incidence of kidney stones in children evaluated in the emergency department. J Pediatr. 2010; 157:132-7. 10.1016/j.jpeds.2010.02.004 20362300

[3] 3. Dwyer ME, Krambeck AE, Bergstralh EJ, Milliner DS, Lieske JC, Rule AD. Temporal trends in incidence of kidney stones among children: a 25-year population based study. J Urol. 2012; 188:247-52. 10.1016/j.juro.2012.03.021 PMC3482509 22595060

[4] 4. Rellum DM, Feitz WF, van Herwaarden AE, Schreuder MF. Pediatric urolithiasis in a non-endemic country: a single center experience from The Netherlands. J Pediatr Urol. 2014; 10:155-61. 10.1016/j.jpurol.2013.07.019 23981680

[5] 5. Sáez-Torres C, Grases F, Rodrigo D, García-Raja AM, Gómez C, Frontera G. Risk factors for urinary stones in healthy schoolchildren with and without a family history of nephrolithiasis. Pediatr Nephrol. 2013; 28:639-45. 10.1007/s00467-012-2368-5 23212561

[6] 6. Penido MG, Srivastava T, Alon US. Pediatric primary urolithiasis: 12-year experience at a Midwestern Children's Hospital. J Urol. 2013; 189:1493-7. 10.1016/j.juro.2012.11.107 23201378

[7] 7. Amancio L, Fedrizzi M, Bresolin NL, Penido MG. Pediatric urolithiasis: experience at a tertiary care pediatric hospital. J Bras Nefrol. 2016; 38:90-8. 10.5935/0101-2800.20160014 27049370

[8] 8. Oakes WJ, Tubbs RS: Chiari malformations. Winn HR (ed) Youmans neurological surgery, third edition, Philadelphia: Elsevier, 2004:3347–3361.

[9] 9. Tasian GE, Copelovitch L. Evaluation and medical management of kidney stones in children. J Urol. 2014; 192:1329-36. 10.1016/j.juro.2014.04.108 24960469

[10] 10. C. Radmayr (Chair), G. Bogaert, H.S. Dogan, J.M. Nijman (Vice-chair), M.S. Silay, R. Stein, S. Tekgül Guidelines Associates: L.A. ‘t Hoen, J. Quaedackers, N. Bhatt. Paediatric Urology. https://uroweb.org/guideline/paediatric-urology/

[11] 11. Bilen CY, Koçak B, Kitirci G, Ozkaya O, Sarikaya S. Percutaneous nephrolithotomy in children: lessons learned in 5 years at a single institution. J Urol. 2007; 177:1867-71. 10.1016/j.juro.2007.01.052 17437838

[12] 12. Zeren S, Satar N, Bayazit Y, Bayazit AK, Payasli K, Ozkeçeli R. Percutaneous nephrolithotomy in the management of pediatric renal calculi. J Endourol. 2002; 16:75-8. 10.1089/089277902753619546 11962558

[13] 13. Shokeir AA, Sheir KZ, El-Nahas AR, El-Assmy AM, Eassa W, El-Kappany HA. Treatment of renal stones in children: a comparison between percutaneous nephrolithotomy and shock wave lithotripsy. J Urol. 2006; 176:706-10. 10.1016/j.juro.2006.03.080 16813924

